# Preface to special topic on AI algorithms and cases: to energize digital economy

**DOI:** 10.1093/nsr/nwad170

**Published:** 2023-06-08

**Authors:** Zongben Xu, Heng Tao Shen, Shi-Min Hu

**Affiliations:** School of Mathematics and Statistics, Xi’an Jiaotong University, China; School of Computer Science and Engineering, University of Electronic Science and Technology of China, China; Department of Computer Science and Technology, Tsinghua University, China

Since the turn of the century, innovations in science and technology have been in full swing across the globe. A new wave of scientific inventions and industrial transformations are constantly reshaping the economic structure of the world. At the forefront of this trend is the digitalization of industries. When combined with a new form of productivity such as artificial intelligence (AI), this wave of scientific and technological revolution is driving digital transformations and adoptions of smart devices throughout society.

To seize the strategic opportunity of the global digital economy, and to demonstrate its leadership, starting from 2022, Pazhou Lab (Huangpu) has been hosting an International Algorithm Case Competition in the Guangdong-Hong Kong-Macau Greater Bay Area (Huangpu) (GBA). This is an effort intended to help the GBA to build a Big Data and AI ecosystem with a nationwide prestige and influence. Based in GBA and aimed at the entire country, the competition invites algorithmic solutions, gathers cutting-edge technology, and attracts top talents worldwide.

Themed as ‘Seeking cases in the Bay, creating algorithms together’, the first competition proposed the ‘knockout’ system and the ‘rating’ system. With 10 high-value topics, the competition attracted participating teams from top universities at home and abroad, establishing a top-level AI competition located in the GBA and achieved national and international exposure. Five of the 10 topics, targeting algorithms in basic research of national strategic needs, focused on key issues in AI and related fields in the post–deep-learning era (i.e. (i) Chinese historical document analysis and recognition, (ii) robust adversarial attack and defense algorithms for deep learning, (iii) tuning algorithms for pre-trained language models, (iv) algorithms for sample selection and label correction and (v) algorithms for singular value decomposition and inverse). Targeting core technologies in mainstream industrial developments, the other five topics concentrated on important sectors such as smart city and smart manufacturing (i.e. (i) character recognition for street-view shop signs, (ii) detection technology for surface defect in industrial products, (iii) algorithms for 3D object detection with roadside cameras, (iv) object recognition of remote sensing imagery and (v) the panoptic scene graph generation challenge), aiming to develop optimal AI algorithms and achieve breakthrough innovations. It's worth mentioning that the competition topic ‘tuning algorithms for pre-trained language’ is a forward-thinking research effort for the AI-driven natural language processing tools, such as ChatGPT, showcasing its visionary and cutting-edge features.

Centering around the topic ‘AI Algorithms and Cases: To Energize Digital Economy’, this special topic includes a news report to briefly introduce the competition and 10 perspective articles to introduce the 10 champion algorithmic solutions, respectively. The news report was written by the competition committee, covering the competition overview, competition structure and achievements [1]. The 10 perspective articles were jointly written by topic providers, championship teams and the competition committee. The five perspective articles from the ‘knockout’ system [2–6] focus on topic rationale, innovative theories, and algorithm introductions; and the other five from the ‘rating’ system [7–11] mainly introduce the practical solutions of competition topics. Each perspective article consists of the background introduction, champion's algorithm, award-winning reasons and future directions.

The competition provides potential breakthroughs in fundamental research in the field of AI and gathers optimal algorithms for solving practical problems. We sincerely hope this special topic will inspire readers from related fields and encourage more participation and attempts in exploring the wonderful world of AI to energize the digital economy.

## References

[1] Zhang H , Xu X. Natl Sci Rev 2023; 10: nwad154. 10.1093/nsr/nwad154 37342319 PMC10278976

[2] Liu C , Jian C, Huang J et al. Natl Sci Rev 2023; 10: nwad115. 10.1093/nsr/nwad115 37292085 PMC10246825

[3] Dong Y , Liu C, Xiang W et al. Natl Sci Rev 2023; 10: nwad087. 10.1093/nsr/nwad087 37304460 PMC10257479

[4] Cao T , Chen L, Zhang D et al. Natl Sci Rev 2023; 10: nwad124. 10.1093/nsr/nwad124 37342318 PMC10278975

[5] Shu J , Yuan X, Meng D. Natl Sci Rev 2023; 10: nwad084. 10.1093/nsr/nwad084 37292084 PMC10246833

[6] Xu C , Xu W, Jing K. Natl Sci Rev 2023; 10: nwad083. 10.1093/nsr/nwad083 37292082 PMC10246826

[7] Tang J , Du W, Wang B et al. Natl Sci Rev 2023; 10: nwad141. 10.1093/nsr/nwad141 37347039 PMC10281498

[8] Guo A , Ma J, Dian R et al. Natl Sci Rev 2023; 10: nwad130. 10.1093/nsr/nwad130 37347038 PMC10281496

[9] Jia J , Shi Y, Qu Y et al. Natl Sci Rev 2023; 10: nwad121. 10.1093/nsr/nwad121 37292083 PMC10246814

[10] Yao Y , Chen T, Bi H et al. Natl Sci Rev 2023; 10: nwad122. 10.1093/nsr/nwad122 37324647 PMC10265956

[11] Yang J , Ma Z, Wang Q et al. Natl Sci Rev 2023; 10: nwad126. 10.1093/nsr/nwad126 37342317 PMC10278980

